# A Metallic Glass-Based Dual-Band-Selective Emitter with Near-Perfect Absorption in Atmospheric Windows

**DOI:** 10.1021/acsami.5c01072

**Published:** 2025-05-21

**Authors:** Tzu-Chieh Hsiao, Wei-Han Wang, Yu-Ching Shih, Sih-Wei Chang, Hsuen-Li Chen

**Affiliations:** † Department of Materials Science and Engineering, 33561 National Taiwan University, Taipei 10617, Taiwan; ‡ Center of Atomic Initiative for New Materials, National Taiwan University, Taipei 10617, Taiwan

**Keywords:** metallic glass, perfect absorber, omnidirectional emission, atmospheric window, dual-band-selective emitter, radiative cooling, asymmetric cavity structure

## Abstract

A new trilayered structure design of metallic glass film was proposed and demonstrated to achieve near-perfect dual-band-selective absorptions within mid-wavelength infrared (MWIR) and long-wavelength infrared (LWIR) bands in atmospheric windows. A metal–insulator–metal (MIM) structure was used as a selective absorber for decades; however, due to the highly reflective properties of metal in the infrared region, the structure was difficult to apply within the mid- and long-infrared region. By applying the metallic glass film as a top layer, the asymmetric metallic glass–insulator–metal (MGIM) structure could display remarkably high absorptance in the mid-IR region, a distinct feature that a conventional metal–insulator–metal (MIM) structure could not achieve. In addition, the MGIM structure exhibited outstanding omnidirectional properties. The fabricated MGIM structure exhibited near-unity absorptions within MWIR and LWIR atmospheric windows, with an absorptance of 96.7% and 98.8% at peak wavelengths of 3.9 and 10.8 μm, respectively. These remarkable characteristics of the AlNiY-based MGIM structure make it highly promising for heat dissipation applications. The MGIM structure showed better radiative cooling performance than typical MIM emitters and an ideal broadband emitter. Furthermore, compared with other dual-band emitters that utilize complex metamaterial structures, the lithography-free process of our approach dramatically reduces the cost and simplifies the processes.

## Graphical abstract

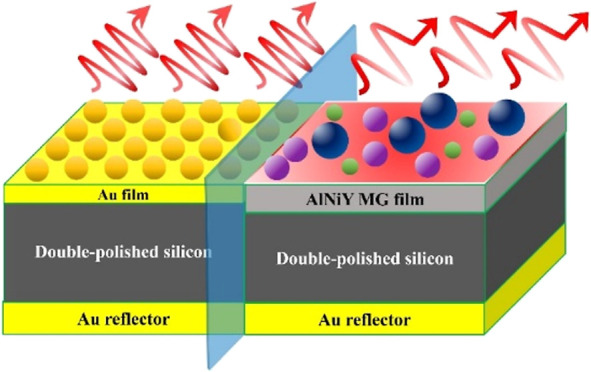

## Introduction

Optical absorbers with wavelength selectivity have garnered enormous attention in recent decades.^1–4^ According to Kirchhoff’s law, they can efficiently emit thermal radiation at desired spectral ranges.^5^ Selective emitters are of significant interest and importance in various applications, including structural colors,^6,7^ thermophotovoltaic (TPV) conversion,^8,9^ gas sensing,^10,11^ thermal camouflage,^12,13^ radiative cooling,^14,15^ and radar stealth technology.^16,17^ Different applications have specific requirements for absorption properties; thus, various optical designs on the choice of materials and structures have been studied and developed. Metamaterials such as plasmonic nanostructures and metallic photonic crystals that induce surface plasmon polaritons (SPPs) have emerged as promising optical absorbers with a smaller dimension than the designed wavelength.^18–21^ By carefully designing the geometric and structural parameters, they can exhibit perfect selective absorptions within a narrow bandwidth at the specific wavelength.^22^ However, these structures typically require complicated fabrication processes such as lithography and etching, which inevitably increases costs and restricts their practical use in large-scale production. On the other hand, optical absorbers composed of multilayered thin films provide a lithography-free approach to achieve selective absorption. By tailoring the layer properties such as thickness and optical constants, the behavior of the incident wave can be manipulated within each layer, inducing interference or resonance at specific wavelengths.^23–25^ Various multilayered structure designs have proven effective and applied, such as Fano resonance,^26^ Tamm plasmon polaritons (TPPs),^27,28^ and metal–insulator–metal (MIM) structures.^29–32^ Through the appropriate design of the multilayered structure, the structure can dissipate heat through thermal radiation, thereby achieving a radiative cooling performance.

Mid-wavelength infrared (MWIR, 3–5 μm) and long-wavelength infrared (LWIR, 8–13 μm) are two major atmospheric windows in the IR region.^33,34^ The Earth’s atmosphere exhibits relatively high transparency within these spectral bands, allowing thermal radiation to pass through with minimal absorption. On the other hand, the Earth’s atmosphere becomes highly absorbed for the wavelengths outside the atmospheric windows. This feature is crucial in various essential applications in the IR region. For example, a low emissivity profile within the MWIR and LWIR atmospheric windows can effectively suppress the thermal signatures of the object. This characteristic prevents the IR cameras from detecting the object, demonstrating infrared camouflage.^13,35,36^ In contrast, selective emissions within MWIR and LWIR bands allow the thermal radiation to provide a passive cooling strategy that does not require extra power consumption.^37^ The Earth has a surface temperature of around 300 K, while the cosmic microwave background of the universe has a thermal radiation spectrum at a temperature of around 3 K. The significant temperature difference between the Earth and the universe can potentially be utilized to cool the object surface below the ambient surroundings by emitting thermal infrared radiation to the universe through the atmosphere.^14^ Radiative coolers with broadband emission cannot cool the temperature significantly below ambient temperature due to their strong absorption of incoming atmospheric thermal radiation outside the transparency window. Nonetheless, radiative coolers with selective emissions through atmospheric windows can effectively isolate and mitigate atmospheric radiation. In other words, the selective thermal emission directly toward outer space leads the coolers to encounter minimal radiative heat exchange with their surroundings. Such an emitter can thus cool down significantly below the ambient temperature.^38^ According to Planck’s law, the peak wavelength of blackbody radiation shifts toward shorter wavelengths as the temperature increases. At lower temperatures, the radiance is primarily concentrated in the LWIR band. However, as the temperature rises, the blackbody radiation peak shifts toward shorter wavelengths, leading to a more significant proportion of radiance in the MWIR band. Consequently, to achieve efficient radiative cooling near room temperature and under high-temperature conditions, such as thermal management of electronic devices, aerospace engineering, and industrial heat dissipation systems, the radiative properties in the MWIR range must also be carefully considered. Therefore, it is possible to attain an outstanding radiative cooling performance by designing the emitter to operate within these two atmospheric windows.

To demonstrate efficient radiative coolers, selective IR emitters with various structures and material designs have been extensively studied.^39^ In particular, different polymer materials are demonstrated to possess emission within the IR range, making them suitable choices for effective radiative coolers.^40^ However, these materials usually either lack near-unity emission within the transparency window or exhibit broad-band emission outside the transparency window. The significant IR absorption outside the transparency window, where the atmosphere is highly emissive, restricts the materials from cooling well below the ambient temperature. Selective emitters utilizing metamaterials were also developed to demonstrate effective radiative cooling performance.^41,42^ Nevertheless, they typically displayed solely selective emission on the LWIR band while exhibiting low emissivity on other spectral ranges. None of these studies have built a selective emitter with dual-band emissions over MWIR and LWIR atmospheric windows. The metamaterial structure must be carefully designed and fabricated to demonstrate dual-band emissions. Liu et al. proposed a dual-band IR emitter based on cross-shaped metallic nanostructures.^43^ It exhibited perfect absorptions at 6.18 and 8.32 μm using alternate geometric parameters of the cross-shaped sublattices. Yao et al. proposed a dual-band IR emitter based on the configuration of photonic crystals.^44^ It displayed near-unity absorptions at the wavelength of around 9 and 11 μm by introducing Si_3_N_4_/Ag- and SiO_2_/Ag-based nanolayers, respectively. It was noted that those emitters cannot exhibit selective emissions directly within the two major atmospheric windows. Building a dual-band emitter with ideally located emissions in the MWIR and LWIR atmospheric windows remains challenging.

Fabry–Pérot emitters are classical cavity structure designs that can achieve selective band emissions.^45,46^ The absorption peaks could reach almost unity by trapping the light in the resonance cavity.^29^ However, owing to the inherent high reflectance of metals at IR wavelengths, Fabry–Pérot emitters were unable to display high absorptance in the LWIR region. The LWIR electromagnetic wave cannot transmit through the top metal layer to ideally induce the Fabry–Pérot resonance. To solve this, people replace the top metal layer with various lossy materials, seeking to determine the effect of the Fabry–Pérot cavity in long wavelength ranges. Kocer et al. proposed a metal–insulator–metal (MIM) structure design based on Cr that could have near-perfect absorption at the wavelength of 1.2 μm.^47^ Xu et al. engineered a VO_2_-based Fabry–Pérot emitter to demonstrate selective absorption in 8 μm to 14 μm.^48^ However, it cannot exhibit high absorption within the MWIR region. Zhang et al. proposed a MIM structure design based on a top metal layer of Ti and a spacer of Ge to exhibit selective absorptions within the MWIR and LWIR bands.^49^ Nevertheless, the top layer thickness was extremely thin, which remained challenging in the deposition process to fabricate such a high-quality thin film. Furthermore, the absorption peaks did not attain unity. As a result, a suitable lossy material as the top layer for the Fabry–Pérot emitter to exhibit perfect absorptions over MWIR and LWIR bands has not yet been found yet.

Metallic glasses, also known as amorphous metallic alloy, have gained enormous attention recently due to their unique physical and chemical properties.^50^ Owing to their amorphous and grain boundary-free structures, metallic glasses possess distinct characteristics such as high hardness and strength,^51^ high plasticity,^52^ excellent corrosion resistance, and wear resistance.^53^ However, bulk metallic glasses (BMGs) fabricated by rapid quenching techniques have limitations of brittleness and large sizes, which restricts their applications in various fields.^54^ Fortunately, the development of thin–film metallic glasses (TFMGs) has overcome the drawbacks associated with BMGs.^55^ TFMGs with a thickness of less than 1 μm can be readily produced using physical vapor deposition (PVD) techniques, including magnetron sputtering, thermal evaporation, and pulsed laser deposition.^56,57^ Among these methods, magnetron sputtering has emerged as the most commonly used method due to its cost effectiveness, scalability, and compatibility. Magnetron sputtering enables the fabrication of high-quality, uniform coatings over extensive areas, typically reaching several square meters,^58–60^ and has been widely adopted in industry for large-area film production. Additionally, it also allows for thin-film deposition on flexible substrates,^61^ making it suitable for applications requiring mechanical adaptability. The deposited films can conform to irregular and non-planar surfaces, extending the applicability of TFMGs to arbitrary geometries. Moreover, TFMGs with diverse properties could be fabricated by adjusting the process parameters such as sputtering power, working pressure, and deposition temperature.^62,63^

This study proposed and demonstrated a new trilayered structure design composed of a metallic glass film to achieve near-perfect dual-band-selective absorptions within the MWIR and LWIR bands. The IR absorptance spectra for different structural configurations of various materials were characterized and analyzed. The proposed asymmetric MGIM structure can exhibit dual-band emissions in the IR region, where two absorption peaks can target well within the MWIR and LWIR bands. Compared with typical MIM structures and ideal broadband emitters, the MGIM structure exhibited an outstanding selective emitting performance. It was suggested that the IR dual-band emitter based on the simple trilayered MGIM structure can be used in radiative cooling and various infrared applications.

## Results and Discussion

### Structure Design for the Dual-Band Emitter within the MWIR and LWIR Bands Based on Metallic Glass Films

The amorphous structure of the metallic glass films exhibited distinct optical properties in the IR region, significantly different from those of conventional metals. To illustrate, Figure 1a shows the schematic description of the atomic arrangement and the electron movements within the traditional metals (Al) and the metallic glass (Al_68_Ni_18_Y_14_). Atoms in Al are arranged in a repeating and periodic pattern (long-range order). Conversely, AlNiY performs a random and disordered atomic arrangement. When applied by an electromagnetic wave, free electrons in Al could move along grain boundaries and interact with the electromagnetic wave. However, free electrons in AlNiY would encounter more collisions and scattering, which decreased the mean free path of the electrons. This phenomenon restricts the electrons from interacting with the electromagnetic wave effectively, resulting in a conduction loss within AlNiY metallic glass films. As a result, the metallic glass would exhibit lossy characteristics distinct from the conventional metals.^64^ The supporting information presented a more detailed discussion of the optical properties of the metallic glass (Figure S1). To illustrate the lossy characteristic of the single-film AlNiY metallic glass, we compared the absorptance spectra of the AlNiY metallic glass film with those of three different conventional metal films, including gold (Au), aluminum (Ni), and nickel (Ni), at varying thicknesses. The results are shown in Figure S2. Due to the limitation of their high reflectance, we can observe that conventional metals cannot achieve significant absorptance in the mid-infrared (MIR) range only when their thicknesses are limited to a few nanometers. In contrast, owing to the lossy nature of the metallic glass, the absorptance can exceed 40% even when the thickness reaches 100 nm.

**Figure 1:**
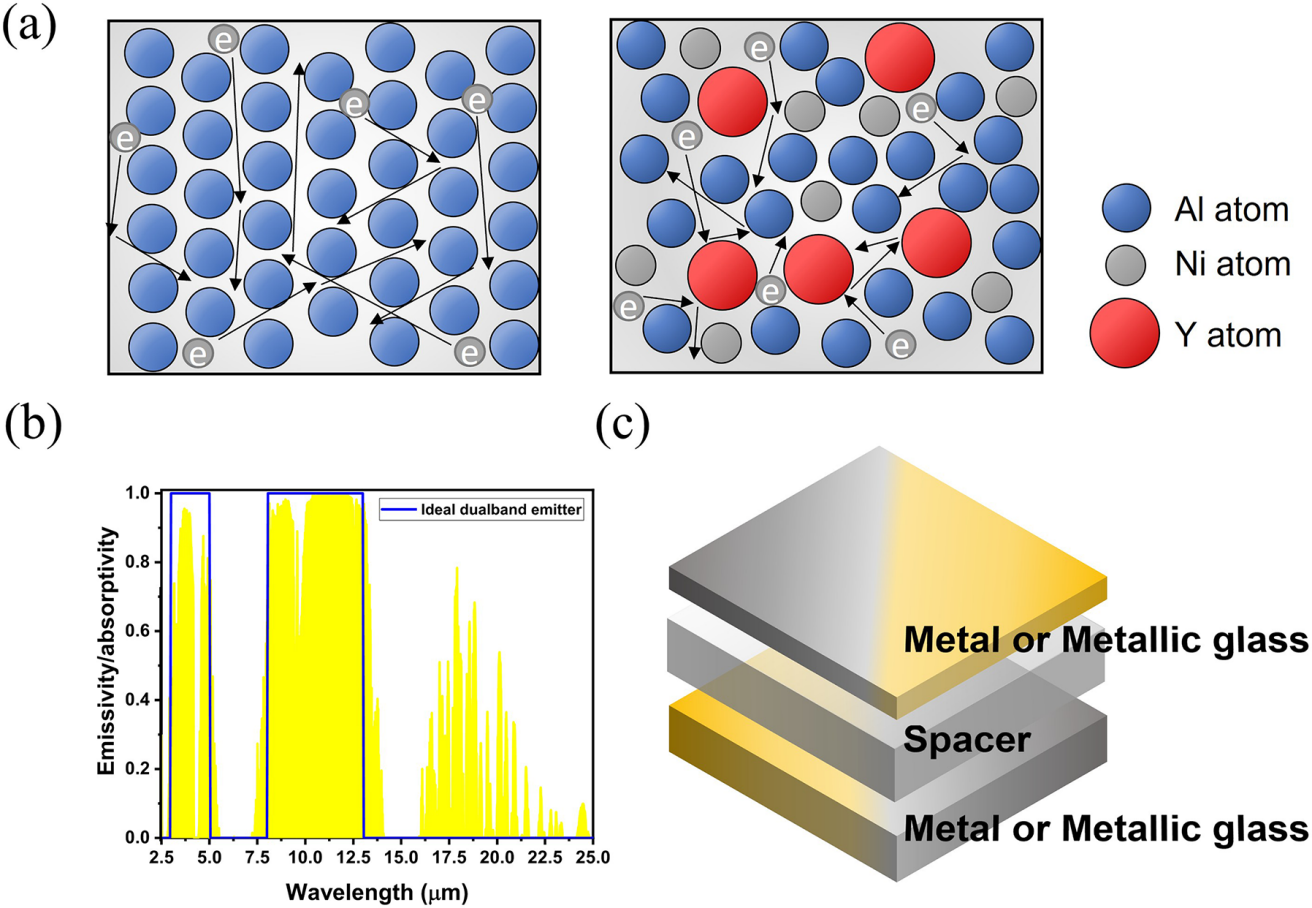
Schematic illustration for the triple-layered structure based on the metallic glass to achieve ideal dual-band absorption within two atmospheric windows. (a) The comparison of the atomic arrangement and the movement of free electrons within the conventional metal (Al) and the metallic glass (AlNiY). (b) The IR absorptance spectrum for the ideal dual-band emitter. The yellow shaded area was the transmittance of the atmosphere. (c) The material design for the triple-layered structure to achieve ideal dual-band absorption.

Leveraging the lossy properties of the metallic glass film, it was expected that the metallic glass could achieve dual-band absorptions within the MWIR and LWIR atmospheric windows by a proper structure design. The emissivity spectrum of an ideal dual-band emitter is shown in Figure 1b. It would emit thermal radiation throughout 3 to 5 μm (MWIR band) and 8 to 13 μm (LWIR band) with near-unity emissivity while being non-radiative outside these wavelength ranges. This feature made the ideal dual-band emitter applicable in versatile uses, including infrared sensing, thermal camouflage, and radiative cooling. For radiative cooling applications, it is important to consider both the MWIR and LWIR bands to achieve efficient radiative cooling across various scenarios. According to Planck’s law, the peak wavelength of blackbody radiation shifts toward shorter wavelengths as temperature increases. Figure S3 illustrates that the radiance is primarily concentrated in the LWIR band at lower temperatures. However, as the temperature increased, the blackbody radiation peak shifted toward shorter wavelengths, leading to a more significant proportion of the radiance in the MWIR band. To further quantify the importance of the MWIR band, we analyzed the total radiance within the MWIR and LWIR atmospheric windows across different temperatures. As shown in Table S1, the increasing radiance fraction indicates that enhanced emissivity in the MWIR band can significantly improve radiative cooling efficiency at elevated temperatures. These findings demonstrate that the ideal dual-band emitter is essential for achieving optimal radiative cooling efficiency, enabling effective thermal management across diverse operating conditions and broadening its potential for various practical applications.

To achieve an ideal dual-band emitter, a new trilayered structure design based on the metallic glass film is demonstrated in Figure 1c. It was composed of two metal or metallic glass layers separated by a spacer, featuring a Fabry–Pérot cavity that exhibited selective absorptions. Traditionally, asymmetric Fabry–Pérot emitters based on the MIM structure were commonly used in the visible-to-near-IR range. However, the high reflectance of the top metal layer in the LWIR region limited the capability of the Fabry–Pérot emitter for LWIR wavelengths. On the other hand, it was possible to extend the resonance wavelength of the asymmetric Fabry–Pérot emitter by applying a metallic glass layer, owing to its unique optical lossy properties. Therefore, optical simulations based on various structure configurations of materials were conducted to achieve dual-band absorptions within two atmospheric windows.

### Characterization for the Trilayered Structures Featuring Various Configurations of Metals and Metallic Glass Films

The absorptance spectra for trilayered structures based on various configurations of conventional metals and metallic glass are shown in Figure 2. The spacer material was silicon (Si) because of its highly transparent properties in the IR region. Two traditional metals, gold (Au) and nickel (Ni), were selected as the metallic layers. Au is a noble metal with highly metallic characteristics, while Ni is often considered a lossy metal. An optical simulation was conducted to attain the dual-band absorptions within two atmospheric windows, which used thickness optimization to target the absorption peaks at 4 and 11 μm. The bottom layer was opaque to ensure the electromagnetic wave was trapped in the Fabry–Pérot cavity and absorbed. The thicknesses of the spacer and top layer for each structure are listed in Table 1.

**Figure 2:**
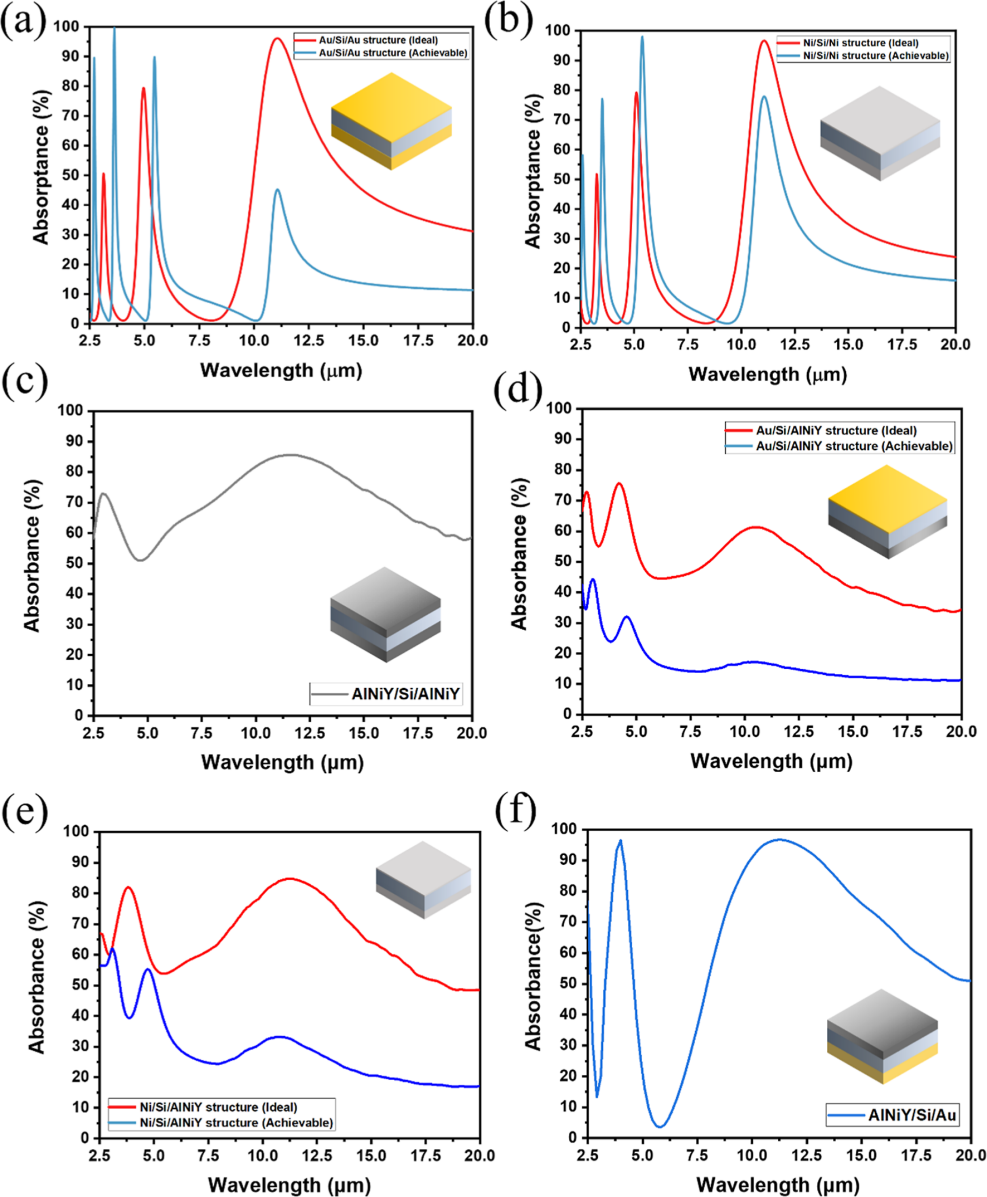
Optical characterization for the triple-layered structures featuring various configurations of conventional metals and metallic glass. (a) Au/Si/Au structure. (b) Ni/Si/Ni structure. (c) AlNiY/Si/AlNiY structure. (d) Au/Si/AlNiY structure with the top layer of Au and the bottom layer of the metallic glass. (e) Ni/Si/AlNiY structure with the top layer of Ni and the bottom layer of the metallic glass. (f) AlNiY/Si/Au structure with the top layer of the metallic glass and the bottom layer of Au. It was noted that the red line denoted the optimized case where the top layer thickness was set at 1 nm.

**Table 1:** Thickness of the Top Layer and Spacer for Trilayered Structures Based on Different Material Configurations

structure configuration	ideal top-layer thickness (nm)	ideal spacer thickness (nm)	achievable top-layer thickness (nm)	spacer thickness (nm)
Au/Si/Au	1.0	1162.0	4.3	1460.0
Ni/Si/Ni	3.9	1196.0	7.3	1341.0
AlNiY/Si/AlNiY	50.0	564.8	50.0	564.8
Au/Si/AlNiY	1.0	969.4	4.3	1215.1
Ni/Si/AlNiY	1.0	770.6	7.3	1202.1
AlNiY/Si/Au	150.0	730.0	150.0	730.0

Figure 2a,b presents the absorptance spectra of typical MIM structures composed of Au and Ni, respectively. It was shown that the Au/Si/Au structure exhibited a high absorptance of approximately 96% at a resonance wavelength of 11.1 μm, with an optimized top layer thickness of 1.0 nm. On the other hand, the Ni/Si/Ni structure displayed an absorptance of nearly 97% with a top layer thickness of 3.9 nm. Such thin-film thickness ensured the IR electromagnetic wave could transmit through the top metallic layer and be trapped in the cavity. Nevertheless, it was noted that the optimized thickness of 1.0 and 3.9 nm was much smaller than the percolation threshold of Au and Ni, which led to discontinuity within thin films. As a result, the condition of the optimized case was unrealistic since the optical properties of the discontinuous metal films differed from those of the bulk metal films in the simulation. The film thickness of Au and Ni to form a continuous film was about 4.3 nm^65^ and 7.3 nm,^66^ respectively. Therefore, we further presented the absorptance spectra of the MIM structures with a top Au layer thickness of 4.3 nm and a top Ni layer thickness of 7.3 nm. The results showed that the Fabry–Pérot resonance was significantly weakened in both the Au/Si/Au structure and Ni/Si/Ni structures. The absorptance decreased to around 45% as the Au thickness increased to 4.3 nm, while the absorptance decreased to about 80% as the Ni thickness increased to 7.3 nm. The Ni/Si/Ni structure maintained a higher absorptance due to the lossy properties of Ni films. Still, the absorptance could not reach near unity in the IR region by applying a conventional MIM structure. Moreover, this structure design could not achieve dual-band-selective absorption in the IR region. More than two absorption peaks for the MIM structures were observed in the spectrum. The high-reflectance characteristics of top-layer metals made the interference behavior of the Fabry–Pérot cavity more complex, leading to more resonance modes in the IR region.

Figure 2c shows the absorptance spectrum of the AlNiY/Si/AlNiY structure. It was observed that the two absorption peaks positioned at 4.0 and 11.1 μm were accurately located at the MWIR and LWIR atmospheric windows, respectively. However, the absorptance corresponding to the two peaks was about 80% and 90%, respectively. The low absorptance values could be attributed to the optical properties of the metallic glass film. An ideal Fabry–Pérot cavity typically requires highly reflective layers that could reflect the light back and forth to achieve near-unity absorption/emission. The lossy properties of the AlNiY metallic glass film constrained it to be highly reflective to induce Fabry–Pérot resonance entirely. As a result, the structure design utilizing the metallic glass–insulator–metallic glass (MGIMG) structure seemed inferior to that of the MIM structure.

In addition to the configuration of MIM and MGIMG structures, we replaced the bottom metallic layer with a metallic glass layer to see if there were improvements in the absorptance spectrum. Figure 2d,e presents the absorptance spectra of the MIMG structure, composed of a bottom AlNiY layer and a top metal layer of Au and Ni, respectively. It was shown that the absorptance at resonance wavelengths was low compared to the MIM structure. Moreover, the bandwidth became more expansive when the metallic glass film replaced the bottom metal layer. As discussed previously, the lossy characteristics of the metallic glass film as a bottom layer would further weaken the effectiveness of the Fabry–Pérot cavity. On the other hand, the high-reflectance characteristics of the top metallic layer restricted the Fabry–Pérot resonance to work in the LWIR region. Consequently, it was speculated that a proper structure design with the configuration of the metallic glass film as a top layer and the metal layer as a bottom layer could effectively extend the working wavelength of the Fabry–Pérot resonance.

Figure 2f shows the absorptance spectrum of the MGIM structure composed of a top AlNiY layer with a thickness of 150 nm, a Si spacer with a thickness of 730 nm, and a bottom Au layer. It was shown that two absorption peaks were accurately located at the MWIR and LWIR atmospheric windows. The resonance peaks were positioned at 3.9 μm and 10.9 μm, with the absorptance of 97% and 98%, respectively. The results revealed that the MGIM structure could achieve near-perfect dual-band-selective absorptions that resembled the ideal dual-band emitter. The top metallic layer could enable the IR electromagnetic wave to penetrate through the spacer. In contrast, the bottom metal layer was highly reflective to ensure the wave could reflect into the cavity. To summarize the high-emissivity property of the MGIM structure, the peak emissivity and average emissivity within two atmospheric windows (MWIR, 3-5 μm, and LWIR, 8-13 μm) of the MGIM structure and MIM structures have been shown in Table S2.

### Angular Dispersion of the MGIM Structure Based on Different Spacer Materials

Figure 3a,b demonstrates the IR absorptance spectra of the MGIM structure as a function of the incident angle with the spacer material of Si. It was observed that the emitter with a Si spacer exhibited excellent angle insensitivity and omnidirectional properties for both TE and TM waves. The absorption peaks maintained their positions and high values until the incident angle reached approximately 70°. This was unusual since conventional multilayered emitters relying on interference effects typically exhibited strong angular dispersions as the effective spacer thickness would change as the incident angle changed. The great omnidirectional property of the emitter could be attributed to the optical property of the spacer (Si). It was noticed that Si had a higher refractive index (n) compared to common dielectric materials in the IR region. According to Snell’s law, an incident light passing through a medium with higher n values would lead to a smaller refractive angle. As a result, the effective spacer thickness corresponding to incident angles would not vary significantly, which resulted in the enhanced angle tolerance of the Si-based emitter.

**Figure 3:**
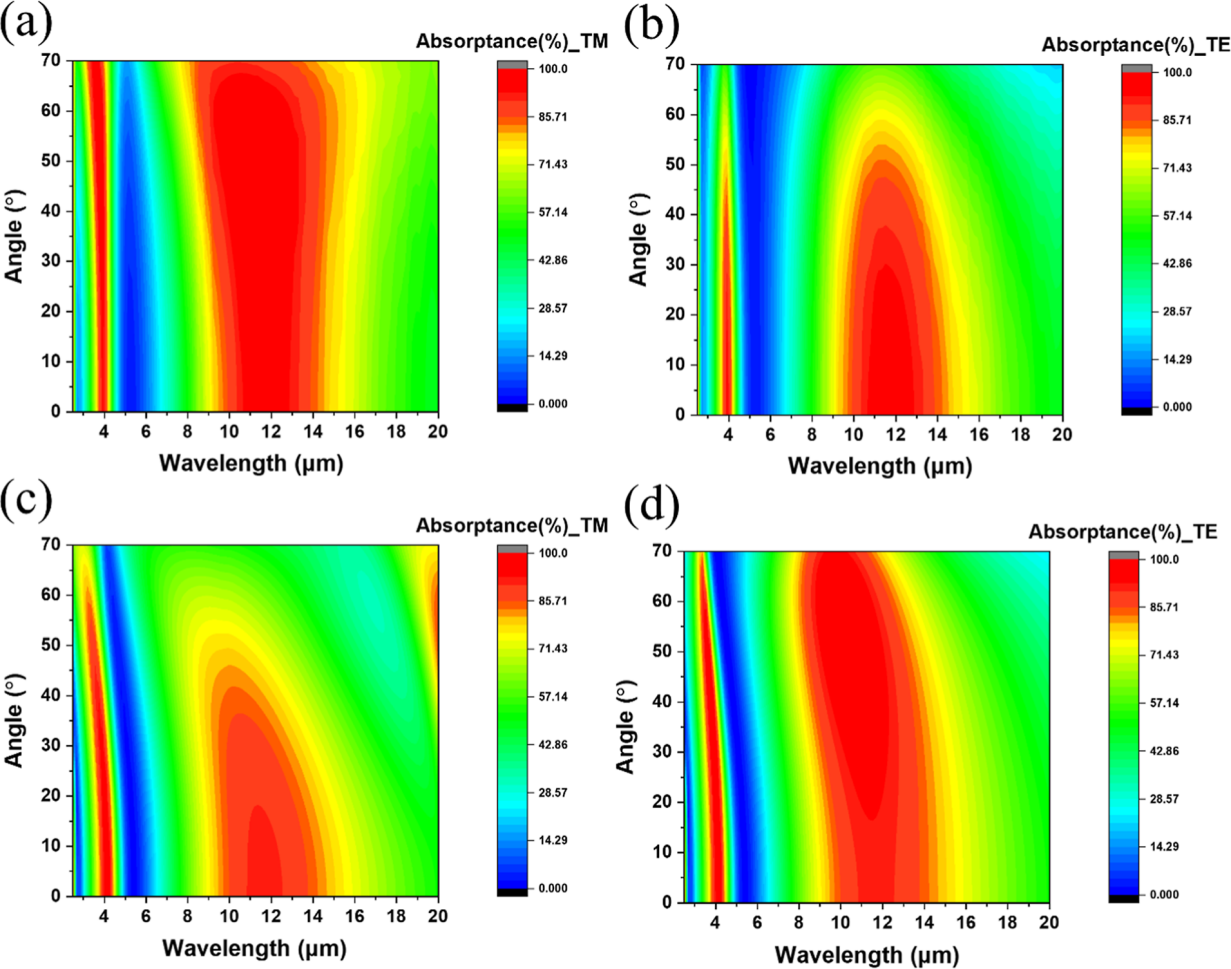
Angular dispersion of the MGIM structure based on different spacer materials. (a,b) TM and TE waves for the Si spacer. (c,d) TM and TE waves for the CaF_2_ spacer.

Figure 3c,d shows the MGIM structure with the spacer material of CaF_2_. Unlike the Si-based emitter, the emitter with a CaF_2_ spacer displayed a strong angular dispersion owing to its low refractive index. The absorption peaks shifted to shorter wavelengths as the incident angle increased. At the incident angle of 50°, the resonance peak of the TE wave shifted from 10.9 μm to 9.4 μm, while the resonance peak of the TM wave shifted from 10.9 μm to 10.1 μm. The results demonstrated that the MGIM structure based on a spacer with a higher refractive index could exhibit selective absorptions with excellent angle tolerance and omnidirectional properties for both MWIR and LWIR bands, a valuable trait that conventional selective emitters cannot compete with the MGIM structure. In addition, the refractive index also influenced the optimized thickness of the MGIM emitter. Figure S4 shows the absorptance spectrum of the MGIM structure with different spacer materials (Si and CaF_2_). It can be observed that as the spacer thickness increased, the resonance peaks of both MGIM structures shifted to longer wavelengths. This shift was particularly noticeable in the MGIM structure with a Si spacer due to its higher refractive index, which allowed interference to occur at a smaller thickness.

### Absorption/Emission Mechanism of the MGIM Structure

To clarify the absorbing mechanism of the MGIM structure and the conventional MIM structure, the electric field amplitude and the absorbed power for AlNiY-based and Au-based selective emitters at the resonant absorption peaks were simulated. Figure 4a presents the absorptance spectrum of the AlNiY-based selective emitter. The gray dotted line depicts the peak position of the fundamental resonance mode at 11.1 μm. Figure 4b,c displays the electric field amplitude profile and absorbed power distribution, respectively. It was observed that a high electric field was applied near the interface between the AlNiY film and the Si layer. This phenomenon differed from the typical Fabry–Pérot emitter, where the high electric field magnitude was confined at the center of the dielectric spacer. The MGIM structure is a highly asymmetric Fabry–Pérot emitter that induced non-ideal resonance. The lossy nature of the AlNiY top layer would induce an asymmetric interference effect that is significantly different from the ideal Fabry–Pérot resonance in a metal-based MIM structure. Therefore, we noticed that the amplitude of the electric field near the surface is high. Furthermore, it was found that the transmitted light was absorbed mainly by the top AlNiY layer, with minimal absorption within the bottom Au layer. The excess optical loss within the top metallic glass layer contributed to the high electric field-induced absorption in the metallic glass layer.

**Figure 4:**
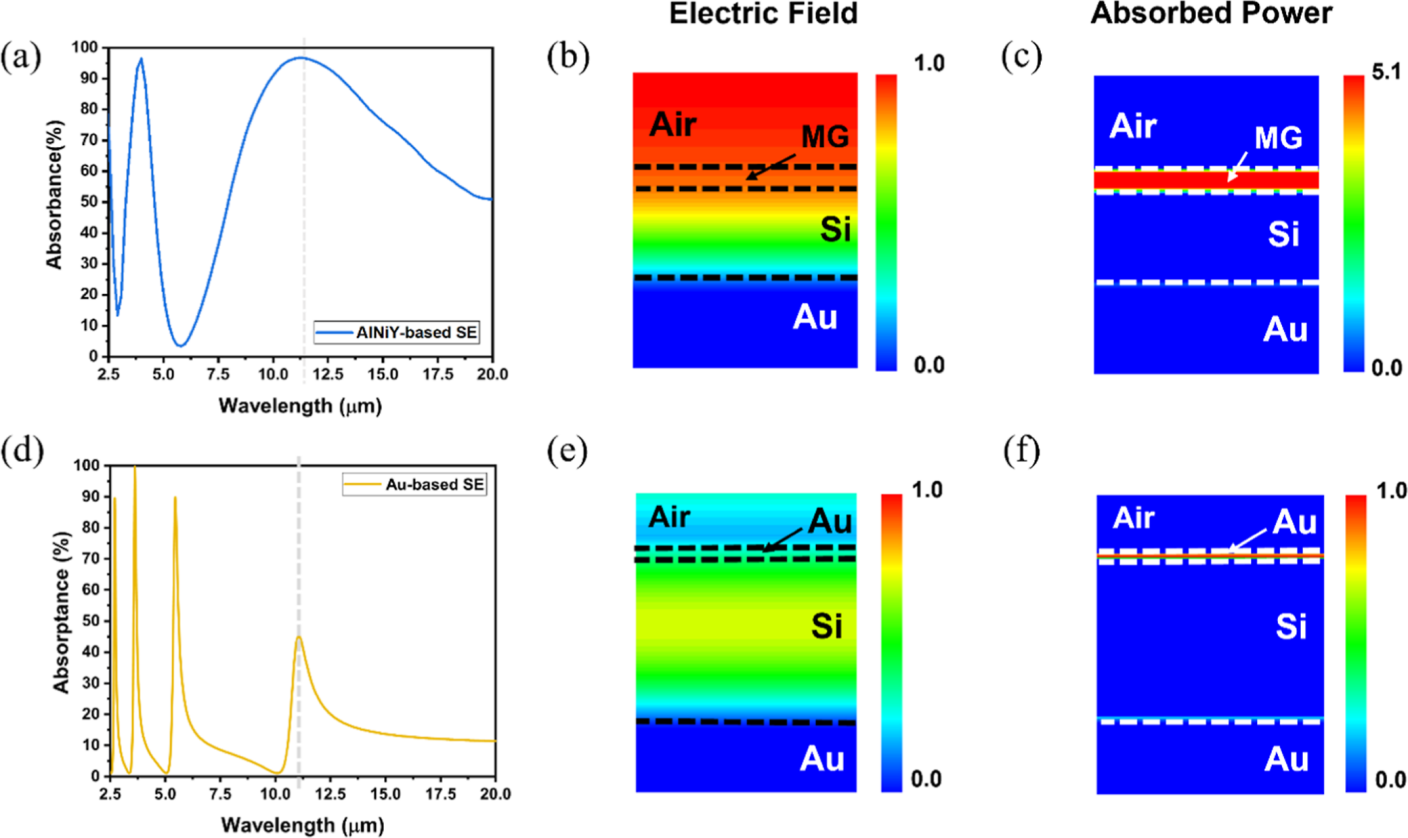
Absorption mechanism for the MGIM structure and conventional MIM structure. (a) The absorptance spectrum of the AlNiY/Si/Au structure. The light-gray dotted line denoted the resonant absorption peak at 11.1 μm. (b,c) The electric field amplitude and the absorbed power distribution of the AlNiY/Si/Au structure at the resonant absorption peak. (d) The absorptance spectrum of the Au/Si/Au structure. The light-gray dotted line denoted the resonant absorption peak at 11.1 μm. (e,f) The electric field amplitude and the absorbed power distribution of the MIM structure at the resonant absorption peak.

To make a clear comparison, Figure 4d–f shows the Au-based emitter’s absorptance spectrum, electric field, and absorbed power distribution, respectively. It was observed that the electric field was confined at the center of the dielectric spacer, revealing that the light was highly trapped in the cavity. However, the amplitude of the electric field for the Au-based emitter was much weaker than that of the AlNiY-based emitter. This property indicated that the typical MIM structure could not induce resonance effectively in the LWIR region, which correlated well with the lower absorptance value in Figure 4d.

### Characterization of the MGIM Structure

Furthermore, an AlNiY-based selective emitter was fabricated to achieve effective radiative cooling. The resonance wavelengths were targeted at 4.0 and 11.0 μm, and the thickness of each layer was determined based on the optimized results. Figure 5a shows the SEM cross-sectional view of the as-fabricated selective emitter. The bottom Au layer was thick enough to be considered an opaque and highly reflective film. The thickness of the Si layer was around 719.3 nm, and the thickness of the top AlNiY layer was approximately 157.4 nm. Figure 5b presents the measured and simulated absorptance spectra of the selective emitter of the wavelengths from 2.5 to 20 μm. Two absorption peaks were observed at around 3.9 μm and 10.8 μm, with absorptance values approaching 96.7% and 98.8%, respectively. The experimental results demonstrated that the AlNiY-based selective emitter could achieve dual-band absorptions within MWIR and LWIR atmospheric windows. However, it was noted that there was a slight discrepancy between the measured and simulated results, which was probably due to the fluctuation of experimental processes. Nevertheless, apart from this deviation, the simulation results agreed well with the measured results. Moreover, the stability of the MGIM-selective emitter is demonstrated in Figure S5. The sample was placed in an environment under the humidity of 50–60% for eight and 18 months, after which its absorptance spectra were re-measured. The absorptance spectra of the emitter showed negligible changes, indicating that the MGIM structure exhibited an outstanding stability.

**Figure 5:**
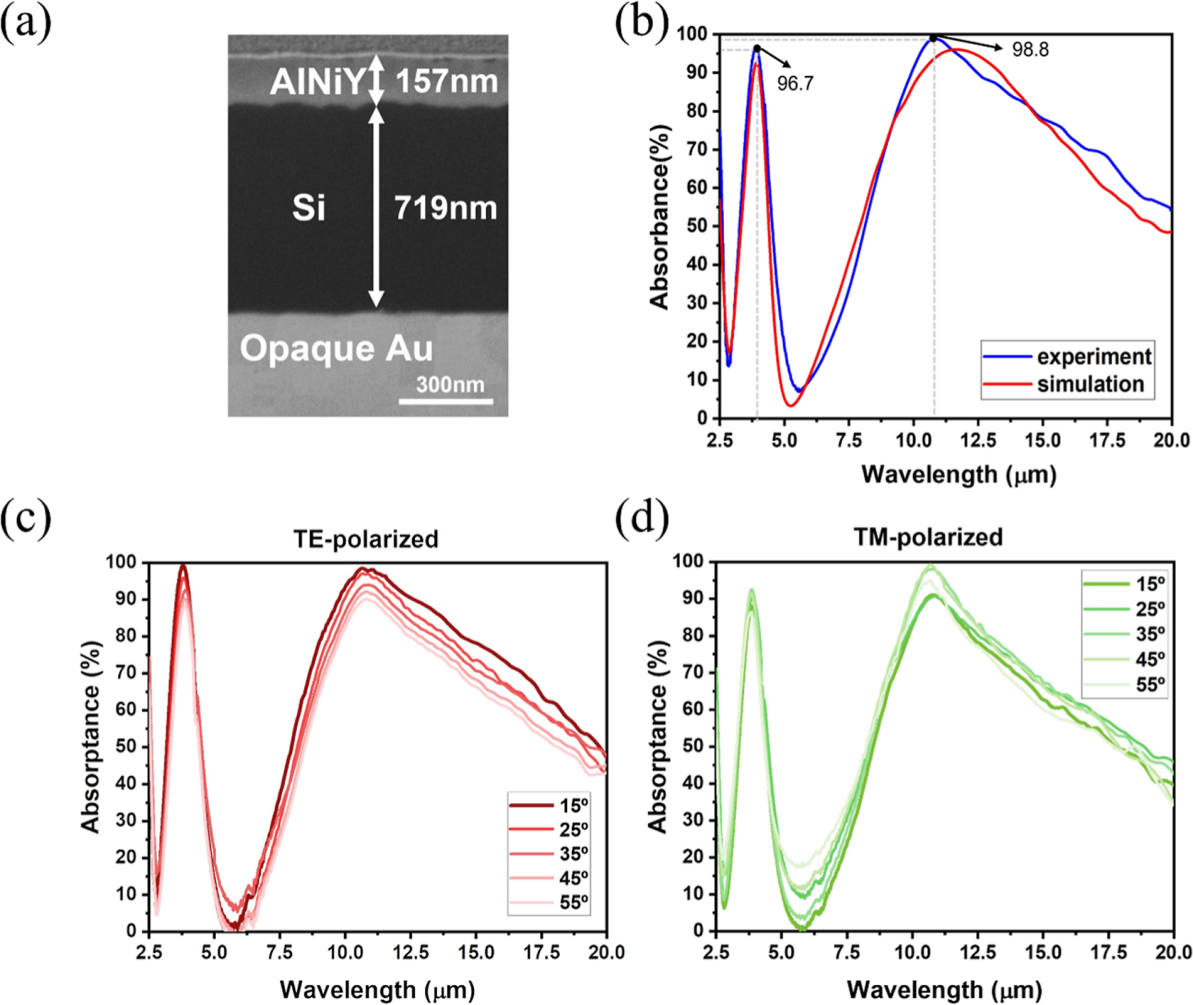
Characterization of the as-fabricated AlNiY-based selective emitter. (a) The SEM cross-sectional image of the AlNiY-based emitter. (b) The measured and simulated IR absorptance spectrum of the AlNiY-based emitter. (c,d) The measured TE- and TM-polarized absorptance spectrum of the AlNiY-based selective emitter at different incident angles.

In addition, Figure 5c,d displays the measured absorptance spectra of the AlNiY-based selective emitter for TE- and TM-polarized waves at various incident angles, ranging from 15° to 55°. It was observed that the positions of the absorption peaks remained consistent regardless of the incident angle, which aligned closely with the simulation results, as discussed before. The results demonstrated the omnidirectional properties of the selective emitter.

Furthermore, magnetron sputtering enables the fabrication of high-quality, uniform coatings over extensive areas, typically reaching several square meters, and has been widely adopted in industry for large-area film production. Additionally, this technology enables thin-film deposition on flexible substrates and allows the deposited flexible films to conform to irregular surfaces and non-planar surfaces, expanding its potential for advanced applications such as thermal management in large area complex geometries and flexible electronic devices.

### Radiative Cooling Performance of the MGIM Structure

Since the MWIR and LWIR bands refer to two major atmospheric windows, the emitter with dual-band-selective absorptions within these two infrared bands will exhibit excellent radiative cooling performance. To characterize the radiative cooling capability of the AlNiY-based MGIM-selective emitter, we calculated the net cooling power (*P*_net_) of the AlNiY-based selective emitter and compared the results with those of the ideal broadband, ideal dual-band, and Au-based emitters. The performance of the radiative coolers was highly related to their spectral emissivity profiles. An ideal broadband emitter would display emissivity like a blackbody within the emission band with wavelengths greater than 4 μm. An ideal dual-band-selective emitter would display near-unity emissivity within two atmospheric windows ranging from 3 μm to 5 μm and 8 μm to 13 μm. The IR emissivity spectra of the four types of emitters are shown in Figure 6a.

**Figure 6:**
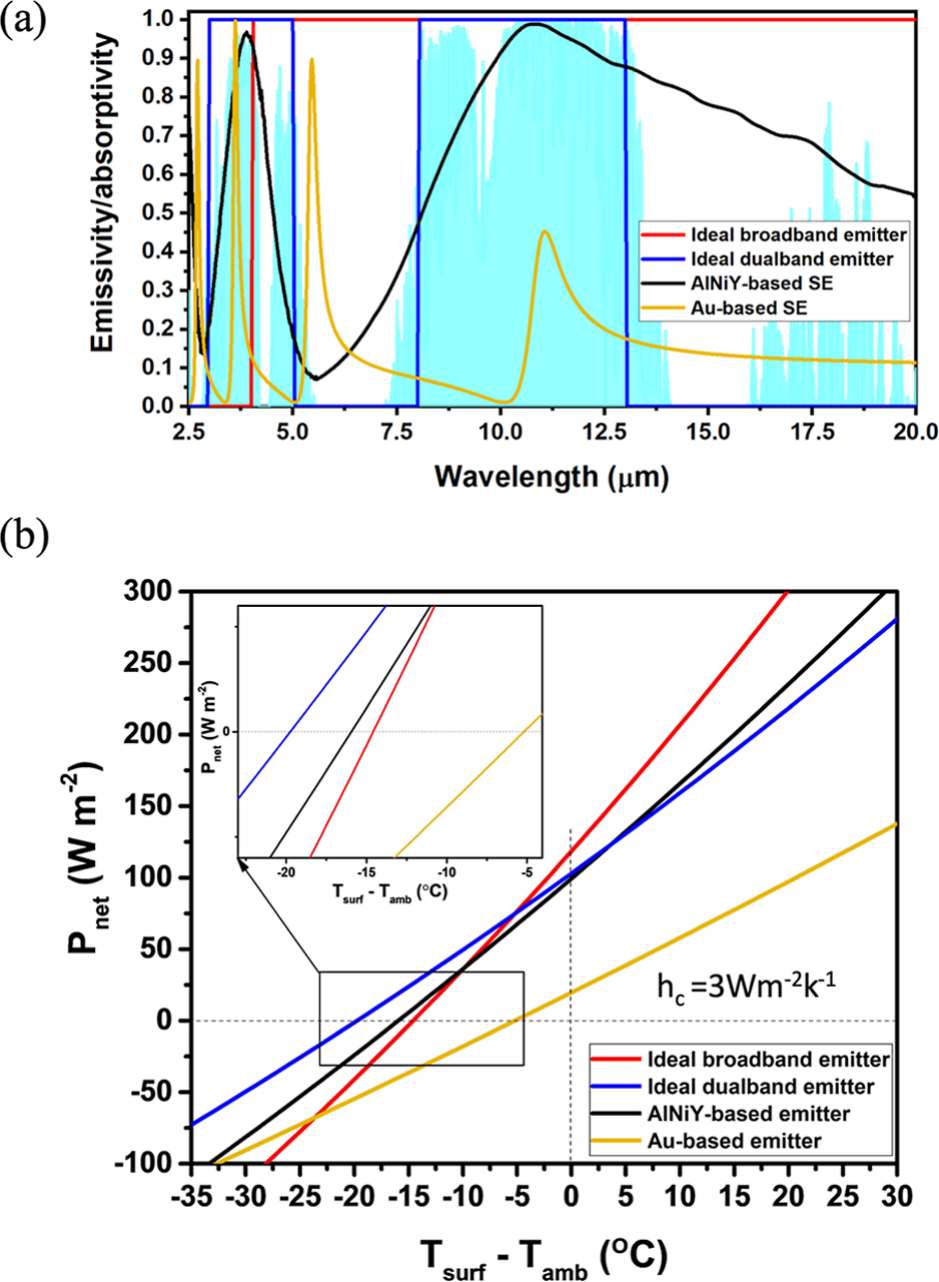
Characterization of the radiative cooling ability of the as-fabricated AlNiY-based selective emitter. (a) The emissivity spectrum of different types of emitters. The light-blue graded area represented the atmospheric transmittance. (b) The net cooling power of the emitters with *h*_c_ = 3 W m^–2^ K^–1^.

The net cooling power of a radiative cooler (*P*_net_) could be described as follows

1 $$P_{net}=P_{rad}-P_{atm}-P_{con}$$

where *P*_rad_ is the radiative power emitted by the cooler; *P*_atm_ is the absorbed atmospheric radiation power by the cooler; and *P*_con_ = *h*_c_(*T*_amb_ – *T*) is the non-radiative heat gain of the cooler with the surrounding medium, where *h*_c_ is the combined non-radiative heat coefficient.

Figure 6b presents the calculated cooling power for each emitter at an *h*_c_ value of 3 W m^–2^ K^–1^. It was observed that when the temperature of the surface equaled that of the ambient surroundings, the net cooling power for all emitters was positive. This indicated that the emitter would continue to emit energy and lower its temperature to achieve thermal equilibrium. The Au-based emitter exhibited the poorest radiative cooling performance among the four emitters. The net cooling power of the Au-based MIM emitter at room temperature was 19.6 W m^–2^. It appeared to be less effective in lowering the temperature well below the ambient temperature (i.e., a more negative value of *T*_surf_ – *T*_amb_). This was because the Au-based MIM emitter exhibited low absorptance in the LWIR band, while it displayed high absorptance peaking outside the atmospheric window.

As for the ideal broadband emitter, it also exhibited poorer radiative cooling performance compared to the AlNiY-based and ideal dual-band emitters despite a higher net cooling power of 118.4 W m^–2^. It was because it emitted less radiative power than it received from the incoming atmosphere radiation at lower temperatures. On the other hand, the outstanding cooling performance of the ideal dual-band-selective emitter was because it allowed minimal heat exchange with the atmosphere.

For the AlNiY-based emitter, it exhibited a cooling power of 99.4 W m^–2^ at room temperature. It yielded an equilibrium temperature of 282.1 K, with a remarkable temperature reduction of 17 K, under the condition of *h*_c_ = 3 W m^–2^ K^–1^ [Figure 6b]. In contrast, although the ideal broadband emitter displayed a larger cooling power of 118.4 W m^–2^ at room temperature, the equilibrium temperature (*T*_eq_ = 284.4 K) was higher than that of the AlNiY-based emitter.

### An Outdoor Radiative Cooling Test of the MGIM Structure

To experimentally prove our calculated result, the experiment setup and subambient temperature of the AlNiY-based emitter are shown in Figure 7. The radiative cooling performance of our MGIM structure was investigated. The wind speed and humidity during the outdoor experiment were 0.1 mph and 74%, respectively. The sample was positioned in a thermal box (Figure 7a,c) made of insulation foam, which was covered by aluminum foil and sealed with a PE cling wrap to minimize the conduction and convection heat transform. The thermocouples were placed on the back surface of the film and inside the thermal box to detect real-time temperatures of the ambient and sample. Figure 7b,d indicates that our AlNiY-based SE could achieve a temperature reduction of 7.16 K. The discrepancy between our experimental and simulation results primarily arises from differences in the non-radiative heat transfer coefficient (*h*_c_) setting between the idealized simulation and the outdoor experiment conditions. Our initial simulations assumed *h*_c_ = 3 W/m^2^ K, representing an idealized scenario achievable only under well-sealed or vacuum conditions, where non-radiative heat exchange was minimized. However, *h*_c_ is typically higher in practical settings due to imperfect sealing and additional convective heat exchange with the surrounding air.

**Figure 7:**
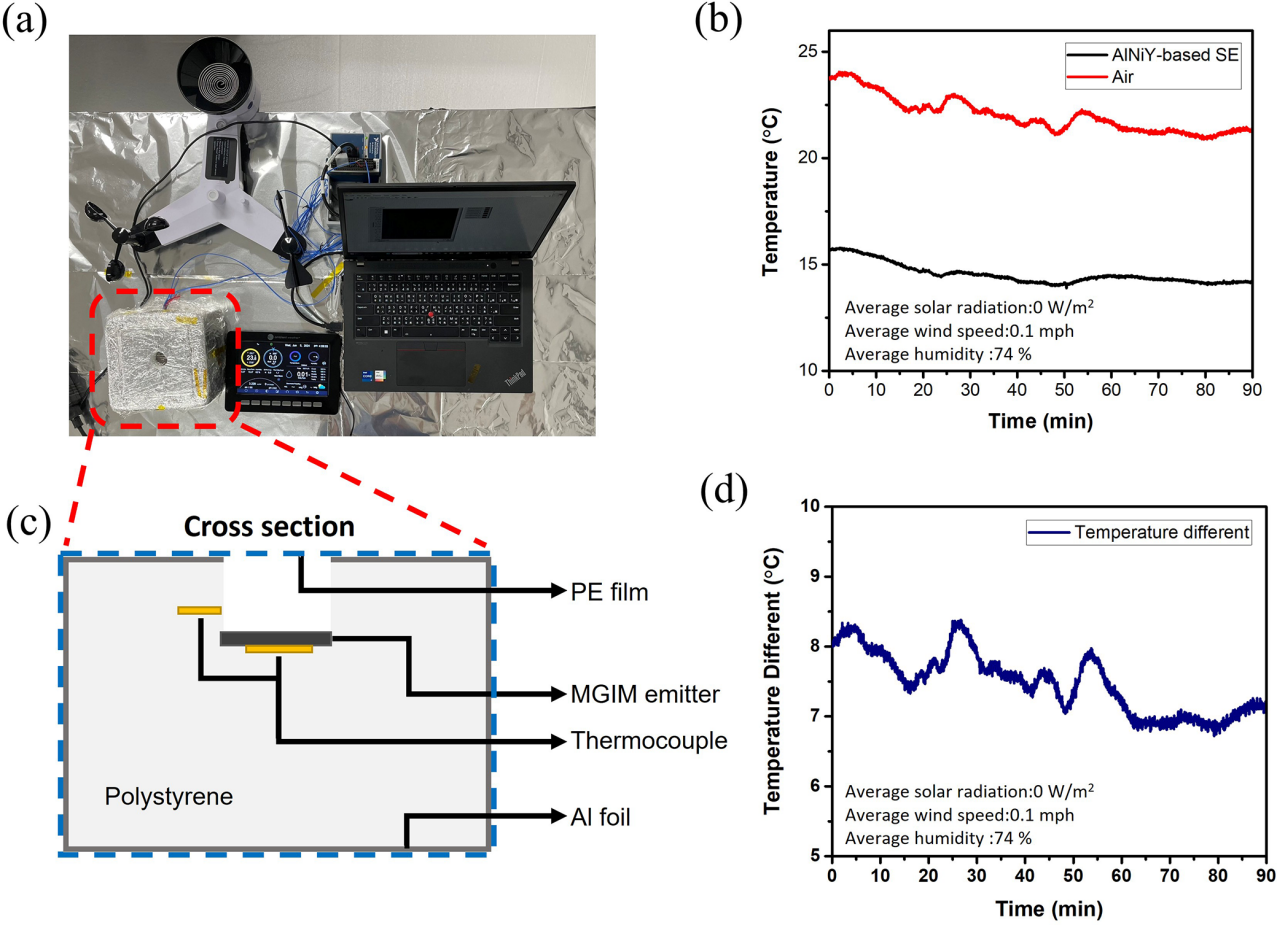
Outdoor radiative cooling test of the AlNiY-based selective emitter. (a) Photograph of the temperature measurement setup. (b) The measured temperature of AlNiY-based selective emitter and air. (c) Cross section of the thermal box. (d) The temperature difference between the AlNiY-based selective emitter and air.

As shown in Figure S6, we found that our experimental result aligns with the calculated temperature reduction at *h*_c_ = 10 W/m^2^ K. This *h*_c_ value is achievable and reasonable under the sealing conditions of our experimental thermal box, and the simulation predicts a temperature reduction of 7.43 K, which closely matches our experimental observation.

The results demonstrated that the AlNiY-based emitter exhibited improved radiative cooling performance compared to those of the Au-based and ideal broadband emitters. The impressive temperature decrease achieved by the MGIM structure has shown its suitability for radiative cooling applications. In contrast to other devices that utilized photonic crystal or metamaterial structures to achieve selective absorption, our emitter achieved selective absorption through a simple deposition process. This lithography-free approach significantly reduced the fabrication cost and simplified the processes.

## Conclusion

In summary, a new trilayered structure design composed of a metallic glass film to achieve near-perfect dual-band-selective absorptions within MWIR and LWIR bands was proposed and demonstrated. The optical properties of the AlNiY metallic glass film were analyzed and studied. The amorphous structure of the metallic glass hindered the movement of electrons drastically, leading to loss properties that conventional metals could not obtain. This distinct feature made the metallic glass suitable for asymmetric cavity structures to induce resonance in both the MWIR and LWIR regions. To showcase the superiority of the metallic glass, the absorptance spectra of different structure configurations based on Au, Ni, and AlNiY metallic glass were simulated and compared. It was shown that typical MIM structures could not achieve near-unity absorption in the LWIR region due to the high-reflectance characteristics of conventional metals. Proper structure design and thickness optimization revealed that the MGIM structure composed of a top metallic glass layer and a bottom metal layer could achieve dual-band absorptions within two infrared atmospheric windows with near-unity absorptance. In addition, the MGIM structure with the spacer material Si exhibited an excellent angular tolerance. The absorption peaks maintained high absorptance as the incident angle changed and did not shift to other wavelengths, a distinct feature that conventional multilayered structures could not obtain. Furthermore, the distribution of the electric field amplitude and the absorbed power revealed that due to its optical loss, the top AlNiY layer would absorb most of the incident light and lead to the increment of absorption.

The experimental results showed that the AlNiY-based emitter could exhibit near-unity absorption peaks positioned at 3.9 and 10.8 μm, which correlated well with the simulation results. By calculating the net cooling power, the AlNiY-based emitter showed superior cooling ability than the Au-based emitter and the ideal broadband emitter, with a net cooling power of 99.4 W m^–2^ at room temperature. At the non-radiative condition of *h*_c_ = 3 W m^–2^ K^–1^, the AlNiY-based emitter could effectively lower the temperature to around 282.1 K, a 17 K decrease below the room temperature. The results of the outdoor experiment confirmed that our AlNiY-based emitter achieved a temperature reduction of 7.16 K, which closely aligns with the predicted value at a non-radiative heat transfer coefficient of 10 W/m^2^ K, demonstrating the effectiveness of our AlNiY-based emitter in radiative cooling applications. Compared to other dual-band emitters based on metamaterials, which require complicated processes, the simple deposition process for the AlNIY-based emitter significantly reduced the fabrication cost and extended the applicability. These outstanding results demonstrated that this new asymmetric MGIM structure could perform in various advanced applications in the MWIR and LWIR regions.

## Experimental Section

### Sample Fabrication

To fabricate the metallic glass–insulator–metal (MGIM)-selective band emitter, a bottom Au layer was deposited onto the silicon (Si) wafer by using DC magnetron sputtering. The sputtering power and the Ar pressure were maintained at 100 W and 2 × 10^–3^ Torr, respectively. Subsequently, the Si spacer was deposited onto the bottom Au layer by using RF magnetron sputtering. The sputtering power was set at 250 W, and the Ar pressure remained at 2 × 10^–3^ Torr. Afterward, an Al_68_Ni_18_Y_14_ metallic glass layer was deposited onto the Si spacer by magnetron sputtering. The sputtering power and Ar pressure were fixed at 150 W and 4 × 10^–3^ Torr, respectively. Before deposition, the chamber was initially evacuated to the pressure of 5 × 10^–6^ Torr. Then, Ar gas was introduced into the chamber at a flow rate of 50 standard cubic centimeters per minute (sccm). During deposition, the substrates were rotated at an average speed of 15 rpm, and the working distance between the target and the sample was 100 mm.

### Characterization

The layer thickness of the MGIM structure was determined by SEM (NOVA 450) cross-sectional views. The optical spectra from the wavelength from 2.5 to 20 μm of the MGIM structures were measured by an FTIR spectrometer (Bruker VERTEX 70) equipped with a deuterated triglycine sulfate (DTGS) detector. The reflectance (*R*) spectra were measured with a Au-coated integrating sphere at the incident angle of 13°. The transmittance (*T*) spectra were measured with zero-angle accessories at normal incidence. The transverse electric (TE)- and transverse magnetic (TM)-polarized waves of the MIR reflectance spectra were measured by using an FTIR spectrometer.

### Simulation

The fitted Lorentz–Drude parameters and the optical constants (*n*, *k*) of the metallic glass films were obtained through an optical thin-film theory. The input data included the measured ellipsometric parameters and reflection and transmission spectra. The simulated optical spectra of the layered structures in the IR region were calculated using the Transfer Matrix Method (TMM).^67,68^ The electric field amplitude and absorbed power in the structures were calculated using a finite-difference time-domain (FDTD) simulation.

### Optimization

We employed an optical thin-film model to refine the structural design in our optimization process. The desired absorption peaks were aligned with the central wavelengths of the two atmospheric windows, with a target high absorptance of 100%, ensuring high emissivity within the MWIR (3–5 μm) and LWIR (8–13 μm) atmospheric windows. The bottom Au layer was selected to be sufficiently thick to provide high reflectivity in the infrared regime, ensuring that the electromagnetic wave can be trapped in the Fabry–Pérot cavity. Additionally, we conducted optical optimization by adjusting the thickness of the top AlNiY thin film and the intermediate Si cavity layer to achieve optimal dual-band absorption.

## Supplementary Material



## Notes

Optical constants of AlNiY, Al, Ni, and Y from 4 μm to 20 μm; absorptance spectrum with different thicknesses of the AlNiY metallic glass, Gold, Aluminum, and Nickel; spectral radiance of a blackbody at the steady-state temperature of 300, 400, 500, 600, and 700; absorptance spectrum of the MGIM structures with different thicknesses and spacer material Si and CaF_2_; absorptance spectra of MGIM structure before and after 8 months, 18 months of stability test; characterization of the radiative cooling ability of the AlNiY-based emitter with different non-radiative heat transfer coefficient (*h*_c_) values; radiance proportion in the 3–5 μm and 8–13 μm atmospheric windows at different temperatures; and peak and average emissivity in the dual band of the MGIM and MIM structures (PDF)
