# Supplementary material for: A Metallic Glass-Based Dual-Band-Selective Emitter with Near-Perfect Absorption in Atmospheric Windows
Source: ACS Appl Mater Interfaces. 2025 May 21;17(22):32181–93. doi: 10.1021/acsami.5c01072 (PMC12147078; doi:10.1021/acsami.5c01072)
Supplement: Supplementary file 1 [file am5c01072_si_001.pdf]

Supporting Information

A Metallic Glass-Based Dual-Band-Selective Emitter with Near-Perfect Absorption in Atmospheric Windows

Tzu-Chieh Hsiao[1], *Wei-Han Wang*[1], *Yu-Ching Shih*[1], *Sih-Wei Chang*[1],

and Hsuen-Li Chen[1,2*]

[1]Department of Materials Science and Engineering, National Taiwan University,

Taipei 10617, Taiwan

[2]Center of Atomic Initiative for New Materials, National Taiwan University, Taipei 10617, Taiwan

* Corresponding author Email: hsuenlichen@ntu.edu.tw

Supporting information

1. Supporting Figures S1

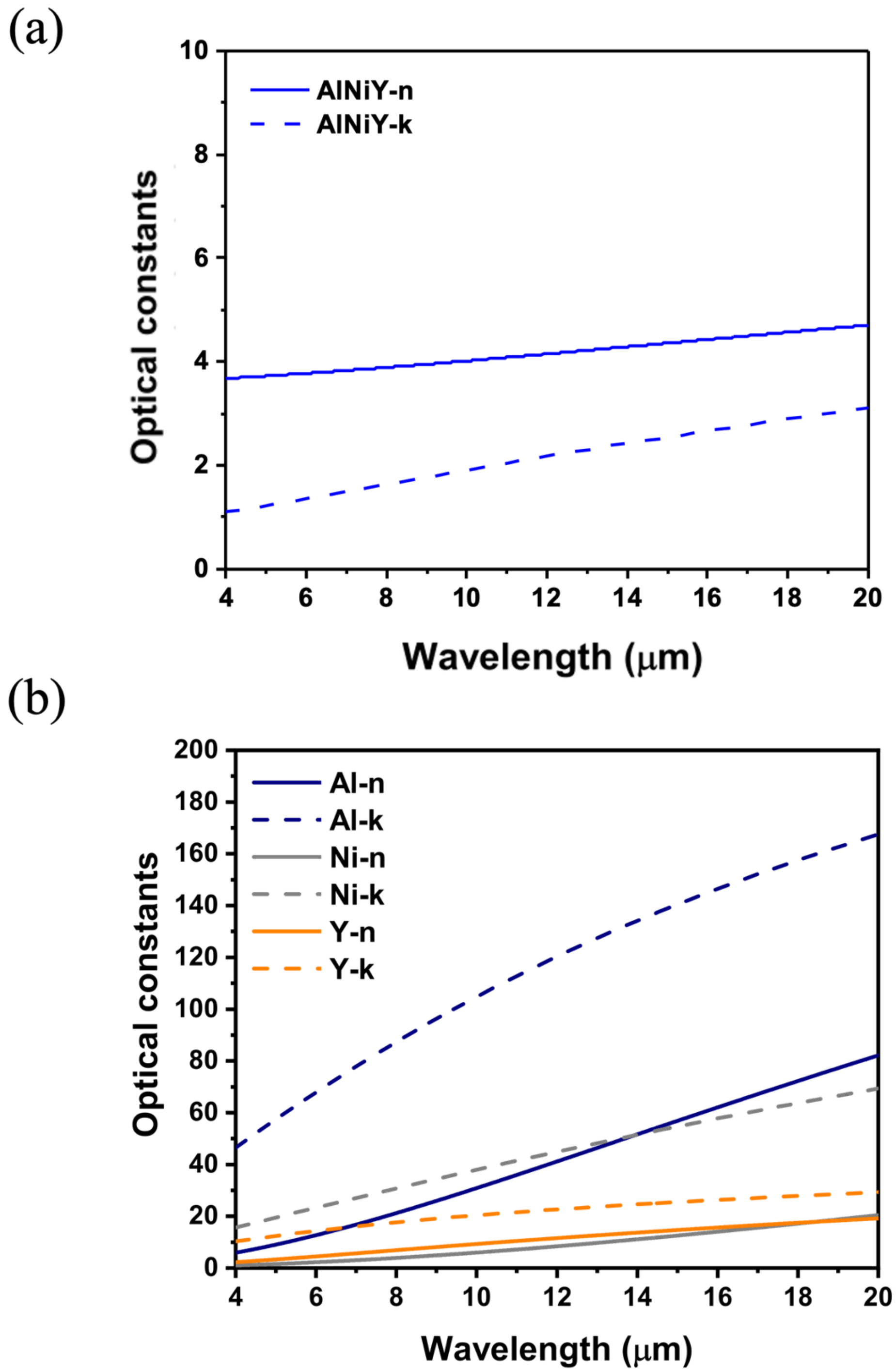

Figure S1 The optical constants of (a) AlNiY (b) Al, Ni and Y from 4 μm to 20 μm.

2. Supporting Figures S2

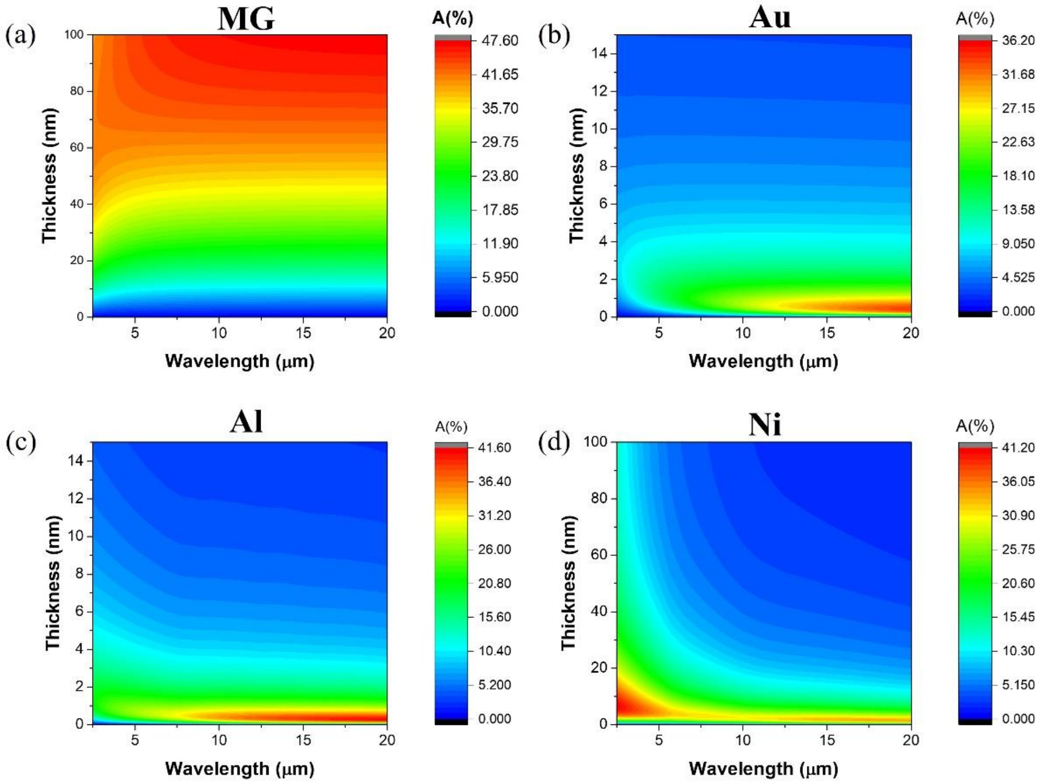

Figure S2 Absorptance spectra with different thicknesses of (a) AlNiY metallic glass, (b) Gold, (c) Aluminum, and (d) Nickel.

3. Supporting Figures S3

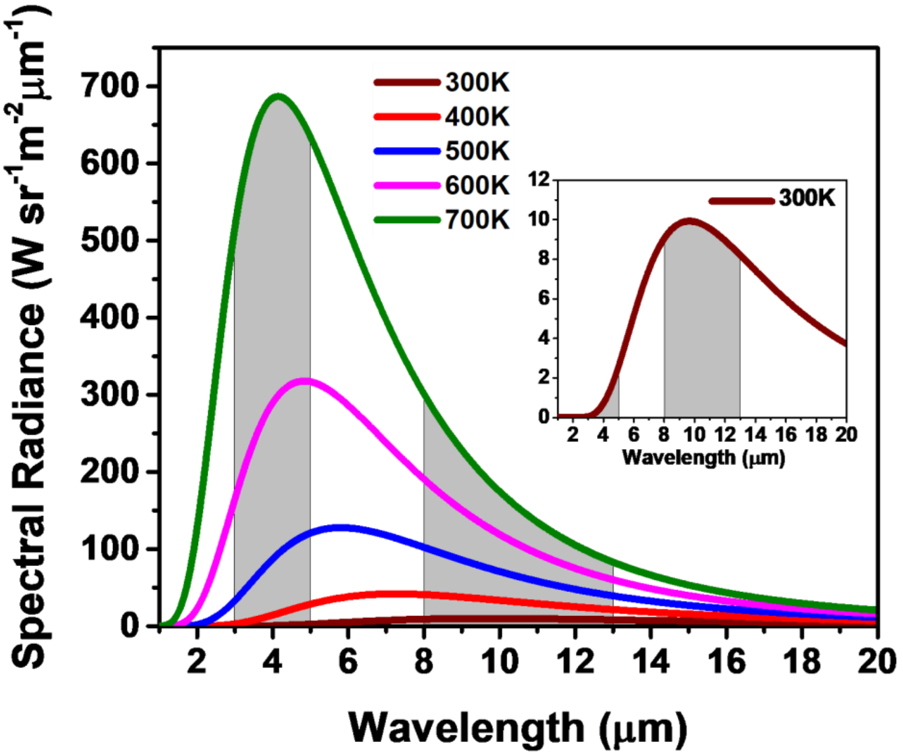

Figure S3 Spectral radiance of a blackbody at the steady state temperature of 300K, 400K, 500K, 600K, and 700K.

4. Supporting Figures S4

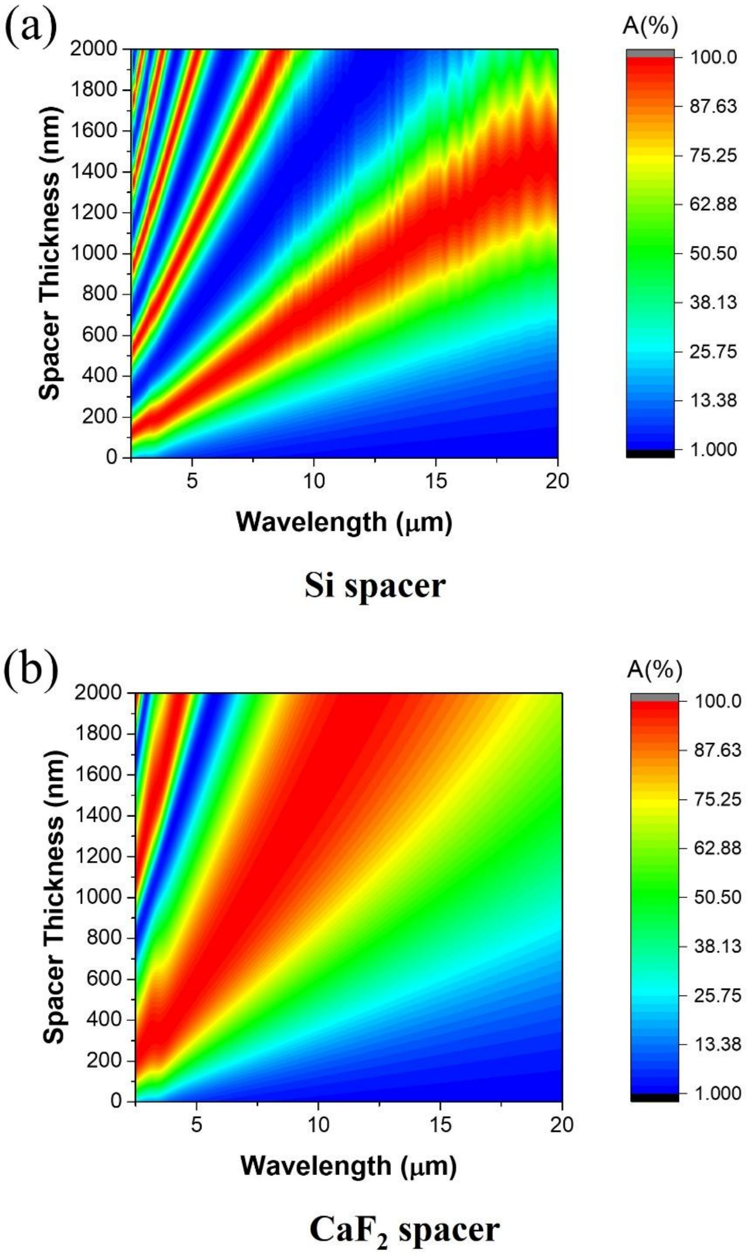

Figure S4 Absorptance spectrum of the MGIM structures with different thicknesses and spacer material (a) Si (b) CaF_2.

5. **Supporting Figures S5**

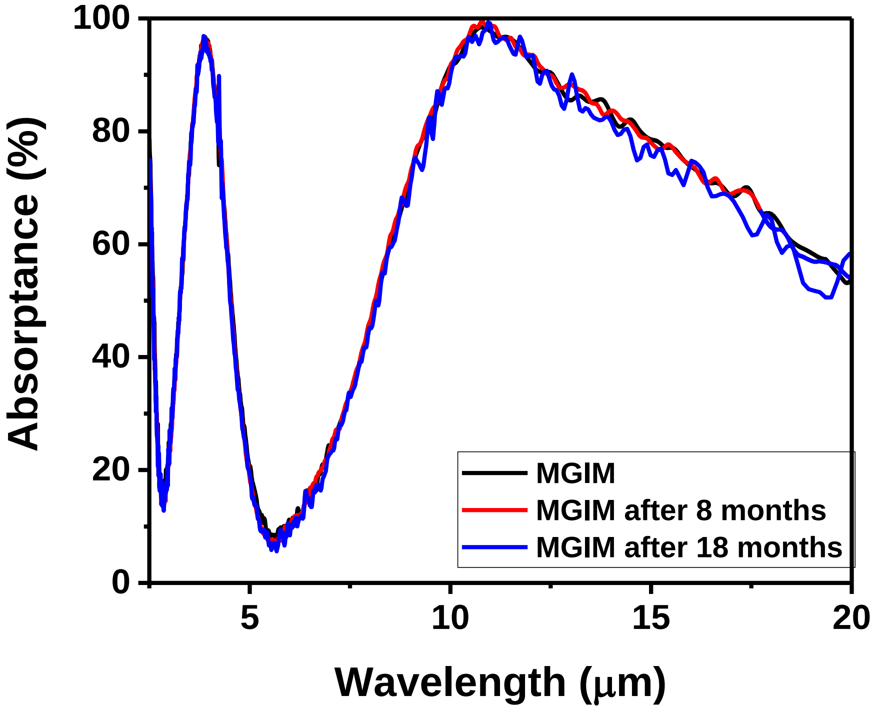

Figure S5 The absorptance spectra of MGIM structure before and after 8 months, 18 months of stability test.

6. Supporting Figures S6

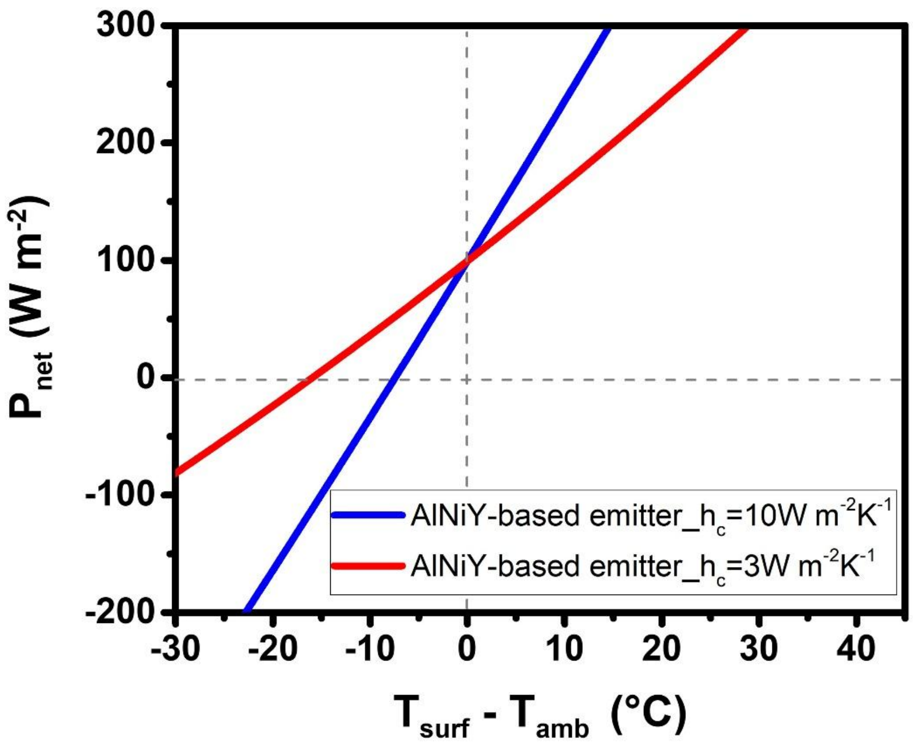

Figure S6 Characterization of the radiative cooling ability of the AlNiY-based emitter with different non-radiative heat transfer coefficient (hc) values.

7. Supporting Table S1

Table S1 Radiance proportion in the 3–5 μm and 8–13 μm atmospheric windows at different temperatures.

Temperature	Radiance Proportion (MWIR)	Radiance Proportion (LWIR)
**300K**	3.85%	96.15%
**400K**	16.02%	83.98%
**500K**	33.22%	66.78%
**600k**	49.23%	50.77%
**700k**	60.85%	39.15%

8. Supporting Table S2

Table S2 The peak and average emissivity in the dual-band of the MGIM and MIM structures.

Structure configuration	Peak emissivity (MWIR/LWIR)	Average emissivity
AlNiY/Si/Au	0.96/0.98	0.76
Au/Si/Au (Ideal)	0.79/0.96	0.44
Au/Si/Au (Achievable)	0.98/0.45	0.15
Ni/Si/Ni (Ideal)	0.79/0.97	0.38
Ni/Si/Ni (Achievable)	0.77/0.79	0.24
